# Selection and validation reference genes for qRT-PCR normalization in different cultivars during fruit ripening and softening of peach (*Prunus persica*)

**DOI:** 10.1038/s41598-021-86755-5

**Published:** 2021-03-31

**Authors:** Shuanghong You, Ke Cao, Changwen Chen, Yong Li, Jinlong Wu, Gengrui Zhu, Weichao Fang, Xinwei Wang, Lirong Wang

**Affiliations:** 1 Fruit Research Institute, Chongqing Academy of Agricultural Sciences, Chongqing, 401329 China; 2 The Key Laboratory of Biology and Genetic Improvement of Horticultural Crops (Fruit Tree Breeding Technology), Ministry of Agriculture, Zhengzhou Fruit Research Institute, Chinese Academy of Agricultural Sciences, Zhengzhou, 450009 China

**Keywords:** Plant biotechnology, Plant development, Plant molecular biology

## Abstract

Quantitative real-time PCR (qRT-PCR) has been emerged as an effective method to explore the gene function and regulatory mechanisms. However, selecting appropriate reference gene (s) is a prerequisite for obtaining accurate qRT-PCR results. Peach is one of important fruit in Rosaceae and is widely cultivated worldwide. In this study, to explore reliable reference gene (s) in peach with different types during fruit ripening and softening (S1–S4), nine candidate reference genes (*EF-1α*, *GAPDH*, *TBP*, *UBC*, *eIF-4α*, *TUB-A*, *TUB-B*, *ACTIN*, and *HIS*) were selected from the whole-genome data. Then, the expression levels of the nine selected genes were detected using qRT-PCR in three peach types, including ‘Hakuho’ (melting type), ‘Xiacui’ (stony hard type), ‘Fantasia’ and ‘NJC108’ (non-melting type) cultivars were detected using qRT-PCR. Four software (geNorm, NormFinder, BestKeeper and RefFinder) were applied to evaluate the expression stability of these candidate reference genes. Gene expression was characterized in different peach types during fruit ripening and softening stages. The overall performance of each candidate in all samples was evaluated. The Actin gene (*ACTIN*) was a suitable reference gene and displayed excellent stability in ‘Total’ set, ‘Hakuho’ samples, S3 and S4 fruit developmental stages. Ubiquitin C gene (*UBC*) showed the best stability in most independent samples, including ‘Fantasia’, ‘NJC108’, S2 sets. Elongation factor-1*α* gene (*EF-1α*) was the most unstable gene across the set of all samples, ‘NJC108’ and S2 sets, while showed the highest stability in ‘Xiacui’ samples. The stability of candidate reference genes was further verified by analyzing the relative expression level of ethylene synthase gene of *Prunus persica *(*PpACS1*) in fruit ripening and softening periods of ‘Hakuho’. Taken together, the results from this study provide a basis for future research on the mining of important functional genes, expression patterns and regulatory mechanisms in peach.

## Introduction

Quantitative real-time PCR (qRT-PCR) is widely used for expression analysis of genes due to its fast, sensitive, specific and accurate characteristics^1,2^. However, the results of qRT-PCR are often affected by many factors, such as RNA concentration, reverse transcription efficiency, amplification efficiency, and experimental process^3,4^. A suitable internal reference gene can eliminate these errors, so the internal reference gene is usually introduced in the qRT-PCR analysis for data correction and standardization^5^. In fact, there is no generality of reference genes in all experiments, and the expression levels of the reference genes in different tissues will change under different treatments^6,7^. Blind use of reference genes to standardize qRT-PCR data will lead to unreliable results, therefore, it is necessary to select appropriate internal reference genes to minimize the distractions.

Previous studies showed that reference genes are always expressed all the time to maintain the basic life activities of cells and their expression levels are less affected by environmental factors^2,8^. Most of the traditional reference genes are the basic components of the cytoskeleton or genes involved in the basic metabolic regulation of the organism^9^. For example, *ACTIN* encodes a cytoskeleton structural protein, *TUB* (β-Tubulin) is mainly involved in cell growth, and *EF-1α* (eukaryotic elongation factor-1*α*) is involved in transcriptional extension. Due to the importance of reference genes in gene expression analysis, many studies on the screening of reference genes with stable expression in higher plants have been conducted and the results showed different reference genes have been applied in different experimental materials and conditions. *ACTINT7* and *TBP* (TATA-box binding protein) are the best reference genes in tripterygium and kumquat^10,11^, *GAPDH* (Glyceraldehyde-3-phosphate dehydrogenase) is the most stable reference gene in all samples in *Primula forbesii*^12^. In *Arabidopsis*, *UBC* (Ubiquitin-conjugating enzyme) is the stable reference gene in seeds, while *UBQ5* (ubiquitin5), *APT1* (adenine phosphoribosyl-transferase 1) and *EF-1α* were the stable reference genes in different tissues^13^. The most stable reference gene of rice in different tissues was *CYC* (cyclophilin)^14^, while in different types of rice samples under drought stress were *UBQ* and *GAPDH*^15^. *PP2A* (protein phosphatase 2A) is the most stable gene under abiotic stress in sorghum^14^. *UBC* is the best reference gene for all samples and different cultivars in *Osmanthus fragrans*, while *ACTIN* is the best reference gene for different flower development stages and different temperature treatments^16^. All these studies showed that the reference genes should be screened, evaluated and verified accurately according to the plant cultivars, different tissues and conditions.

As one popular stone fruit, peach (*Prunus persica* (L.) Batsch) has been known as one of the most common and economically important species worldwide. Recent studies have been payed more attention on the physiological and metabolites changes of peach during fruit development^17–19^, and exogenous treatments on peach ripening and quality in postharvest^20–22^. However, the mechanism of different peach cultivars on peach fruit ripening and softening is still unclear^23^. Biochemical processes occur in a well-defined order under the control of a number of ripening/softening-related genes leading to considerable changes in texture, pigmentation, taste and aroma^24^. Screening suitable reference genes in different peach cultivars at different developmental periods can provide effective help for exploring gene function at molecular level. To our knowledge, only a few reports have been conducted on the suitability of reference gens for normalization of gene expression in peach^25–27^. Limited to the traditional ones published previously in other higher plants, the selected reference genes have been proven to vary significantly across different experimental conditions. Therefore, it is necessary to accurately select the appropriate reference genes in different flesh texture cultivars during peach fruit ripening and softening.

In this study, to validate appropriate reference genes in different cultivars, ‘Hakuho’ (a melting flesh peach genotype, M), ‘Xiacui’ and ‘Fantasia’ (a stony hard flesh peach genotype, SH), and ‘NJC108’ (a non-melting flesh peach genotype, NM) were selected. Nine candidate reference genes: *EF-1α*, *GAPDH*, *TBP*, *UBC*, *eIF-4α* (eukaryotic translation initiation factor 4), *TUB-A* (α-Tubulin), *TUB-B*, *ACTIN*, and *HIS* (Histone) were used to identify the most stable reference genes for normalization from peach whole-genome data. Four software (geNorm, NormFinder, BestKeeper and RefFinder) were used to comprehensively analyze the stability of expression level for these selected reference genes. The results would enrich the reference gene selection in peach, which further improved the stability, repeatability and accuracy of peach gene expression analysis. The suitable reference genes obtained in this study provided a theoretical basis for further exploring the gene expression and regulation mechanisms in peach.

## Results

### Assessment of amplification efficiency and specificity of nine candidate reference genes of peach

**Figure 1:**
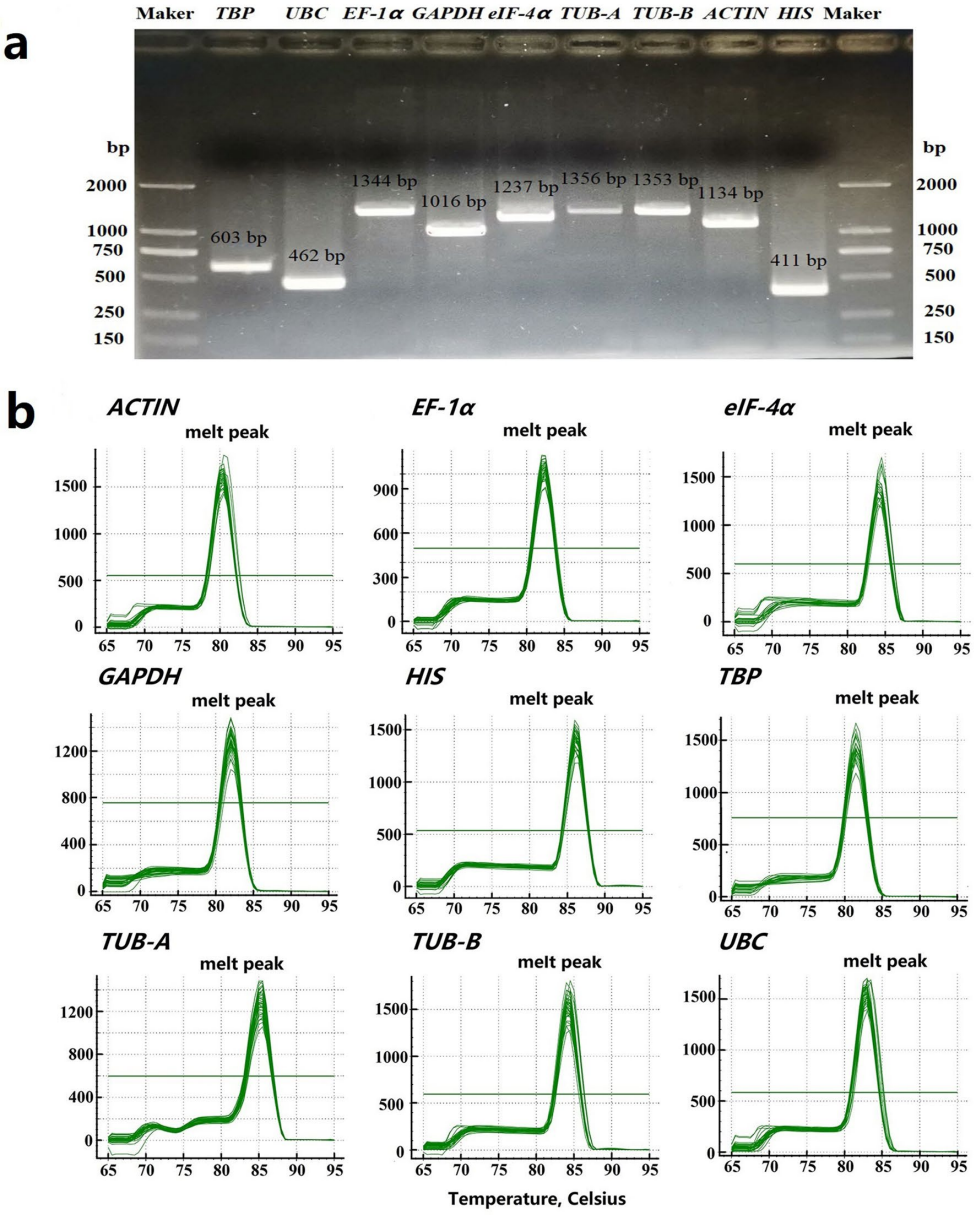
The specificity of primers for PCR amplification. (**a**) Amplification length of nine reference genes in peach by RT-PCR. (**b**) Melting curves generated for nine candidate reference genes by qRT-PCR.

According to the sequences in peach genome, cloning primer pairs of the selected nine candidate reference genes were appropriately designed for PCR amplification (Table S1). The nine candidate genes were successfully cloned and the sequences were confirmed in subsequent experiments (Fig. 1a). Subsequently, qRT-PCR specific primer pairs of the nine genes were further designed, with the amplicon length ranging from 144 to 320 bp. Melting curve analysis of qRT-PCR showed that all the nine selected genes had a single peak, indicating that the amplified products did not have apparent primer dimer or nonspecific amplification (Fig. 1b). The amplification efficiencies (E%) varied from 90.5% to 108% and correlation coefficient (*R*^2^) ranged from 0.992 to 0.998 (Table 1, Fig. S1), which met the standard level (90 < E% < 110; R^2^ > 0.99)^28^.

**Table 1:** Descriptions of candidate reference genes and amplification characteristics for qPCR.

Gene symbol	Gene name	Primer sequence (5′-3′)	Amplicon size (bp)	E%	*R*^2^
*ACTIN*	Actin gene	AGCAGAGCGATTCCGTTGTCC/CCTCCACTCAGCACTATGTTACCAT	153	91.6	0.997
*EF-1a*	Elongation factor -1α gene	GACCAACTGCCTTGCTCCTCTT/CTTGATGAAGTCACGATGTCCAGGT	176	107.5	0.997
*GAPDH*	Glyceraldehyde-3-phosphate gene	GACCAACTGCCTTGCTCCTCTT/GCTCTTCCACCTCTCCAATCCTTAG	144	108	0.994
*TBP*	TATA-box binding protein gene	CCCTTCTGGAATTGTCCCTACTCTC/GCAGTCGTCTTCGGTTCTCTTATTC	162	98.0	0.998
*eIF-4α*	Eukaryotic translation initiation factor 4	TTGGCACAGCAGATTGAGAAGGTT/TCAGGTGGCATTGTAGCAGAGAAC	320	93.1	0.995
*TUB-A*	α-Tubulin gene	GGCTGGTATTCAGGTCGGCAATG/GGTGGAAGAGTTGGCGGTATGTC	233	91.5	0.992
*TUB-B*	β-Tubulin gene	GAGCGAGCAGTTCACAGCCATG/GTTCTCTTCAGCACCGTCCTCCT	193	101.5	0.997
*UBC*	Ubiquitin C gene	GAGTCCTGCTCTCCAGATACGAACT/CGGGTCCATTCCTTTGCTGTTTCA	150	90.5	0.996
*HIS*	Histone gene	GAGTCAAGAAGCCTCACCGTTACC/CGCCTAGCAAGCTGAATGTCCTT	286	95.4	0.993

### Expression levels and variation of the candidate reference genes of peach

**Figure 2:**
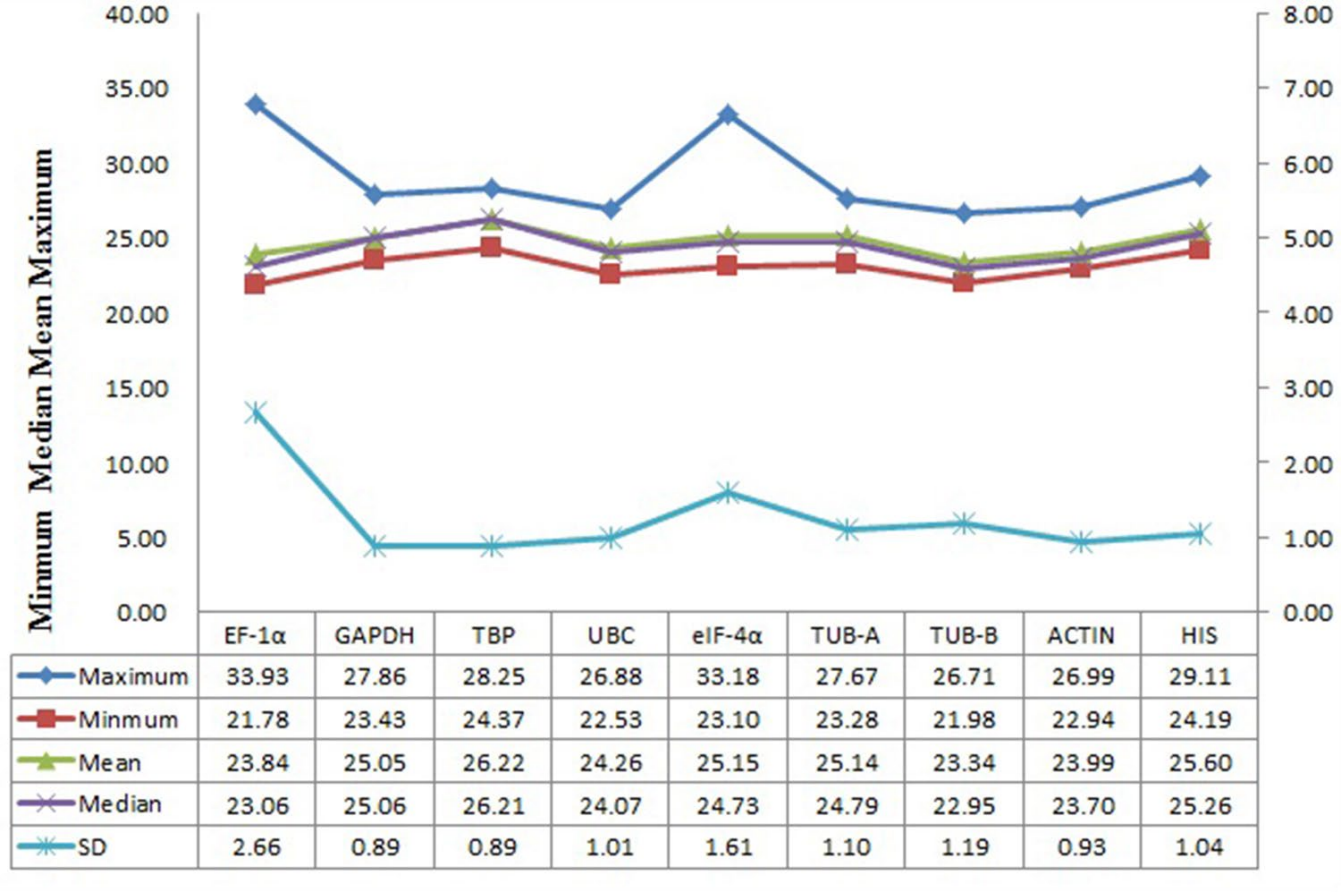
Distribution of the Cq values of nine candidate reference genes across all samples in qRT-PCR analysis. The two ends of the graph represent the maximum and minimum Cq values, respectively. The red line across the graph indicates the mean value.

The expression levels of the nine candidate genes were displayed as Cq values and the raw data were listed in Table S2. A lower Cq value indicates a higher transcriptional expression level of the gene, while a higher Cq value indicates a lower expression level. The nine genes showed different expression levels with great changes of Cq values under experimental conditions (Fig. 2). In general, Cq values of the nine candidate genes ranged from 21.78 (*EF-1α*) to 33.18 (*eIF-4α*), and most Cq values of all test samples were concentrated between 22 and 28. *EF-1α* and *TUB-B* showed higher expression levels with lower Cq values, while *TBP* and *TUB-A* showed lower expression levels with higher Cq values. *EF-1α* had the largest variance (SD = 2.66), *GAPDH* and *TBP* had the smallest variance (SD = 0.89). The Cq value distribution of *ACTIN* was more concentrated than other candidate reference genes.

### Evaluation of expression stability of the reference genes of peach

In the present study, four statistical approaches, geNorm, Normfinder, BestKeeper, and RefFinder were used to evaluate the expression stability of the nine reference genes. The analysis is based on three different data sets: (1) ‘total’, including all experimental samples; (2) samples of different cultivars; (3) samples of different fruit ripening and softening stages.

### geNorm

**Figure 3:**
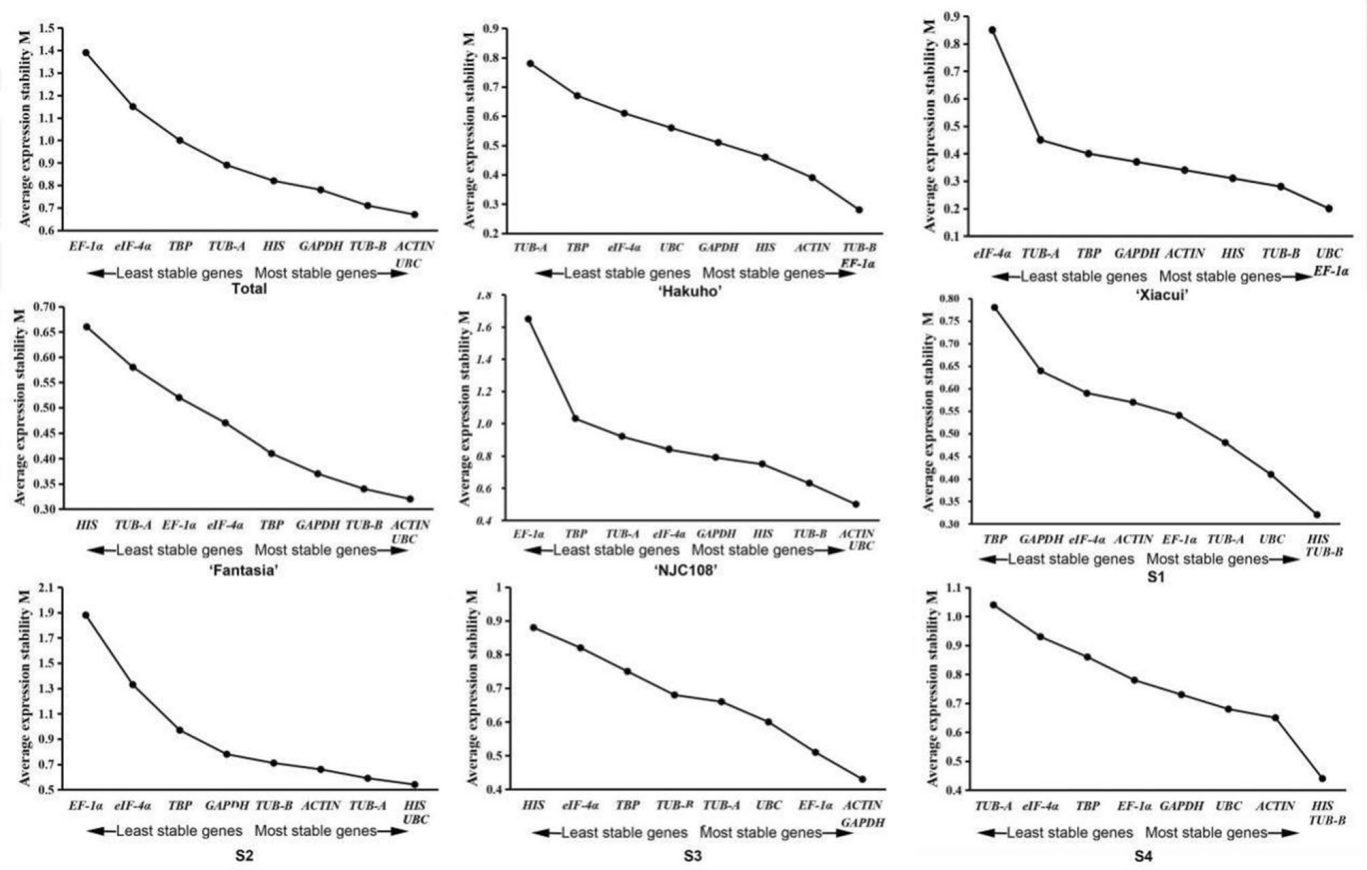
geNorm expression stability with the M values of the nine candidate genes in all sample sets. A lower M value indicates greater stability and the largest value indicates the least stable reference gene.

The geNorm software evaluates the stability of the reference gene by comparing the calculated average expression stability value (M). M-value is negatively correlated with expression stability. Reference gene with the lowest M-value has the highest stability. The software defaults to 1.5 as the critical value. As shown in Fig. 3, the M-values of the nine candidate reference genes were all less than 1.5 in different cultivars and different fruit ripening periods. These results indicated that these genes generally had good stabilities. However, the most stable reference gene (s) is (are) varied in different cultivars and periods. Among the ‘Total’, ‘Fantasia’ and ‘NJC108’, *UBC* and *ACTIN* were the most suitable reference genes; the genes *EF-1 α* and *UBC* were the most stable reference genes in ‘Xiacui’; the genes *EF-1 α* and *TUB-B* were recommended in ‘Hakuho’. During the periods in peach fruit ripening and softening, *TUB-B* and *HIS* were the most stable genes in S1 and S4 stages, while *HIS* and *UBC* were the most stable genes in S2 and the genes *GAPDH* and *ACTIN* were most stable in S3, respectively.

### NormFinder

The NormFinder software ranks the candidate reference genes by the stability value based on both intra- and inter-group variation. The gene with the lowest stability value has the highest stability. In the ‘Total’ group, the stability of the reference genes was ranked as follows: *ACTIN* > *UBC* > *TUB-B* > *GAPDH* > *HIS* > *TUB-A* > *TBP* > *eIF-4α* > *EF-1α* (Table 2). Gene stabilities were also analyzed by different cultivars (Table 3) and different fruit ripening and softening stages (Table 4). The *ACTIN* has the highest stability in S4 and ranks secondly in S1; *UBC* showed the best stability in ‘Xiacui’, ‘Fantasia’, ‘NJC108’ and S2; the gene *EF-1α* ranked first in the S3 but ranked last in the ‘Total’, ‘NJC108’ and S2. According to the analysis of NormFinder, the genes *ACTIN* and *UBC* showed good stability, while the gene *EF-1α* displayed unstable characteristic.

**Table 2:** The stability ranking of candidate reference genes in “Total” analysis by geNorm, NormFinder, BestKeeper and RefFinder.

	Rank	geNorm		NormFinder		BestKeeper			RefFinder	
		Gene	Stability	Gene	Stability value	Gene	SD [± Cq]	CV [%Cq]	Gene	Stability
Total	1	*UBC*	0.67	*ACTIN*	0.23	*ACTIN*	0.66	2.76	*ACTIN*	1.00
	2	*ACTIN*	0.67	*UBC*	0.42	*GAPDH*	0.67	2.68	*UBC*	1.86
	3	*TUB-B*	0.71	*TUB-B*	0.44	*UBC*	0.74	3.05	*GAPDH*	3.36
	4	*GAPDH*	0.78	*GAPDH*	0.64	*TBP*	0.75	2.86	*TUB-B*	3.57
	5	*HIS*	0.82	*HIS*	0.74	*HIS*	0.80	3.14	*HIS*	5.00
	6	*TUB-A*	0.89	*TUB-A*	0.96	*TUB-B*	0.91	3.90	*TBP*	6.09
	7	*TBP*	1.00	*TBP*	1.17	*TUB-A*	0.94	3.72	*TUB-A*	6.24
	8	*eIF-4α*	1.15	*eIF-4α*	1.46	*eIF-4α*	1.09	4.33	*eIF-4α*	8.00
	9	*EF-1α*	1.39	*EF-1α*	2.06	*EF-1α*	1.56	6.53	*EF-1α*	9.00

**Table 3:** The stability ranking of candidate reference genes in different variety of samples by geNorm, NormFinder, BestKeeper and RefFinder.

Variety	Rank	geNorm		NormFinder		BestKeeper			RefFinder	
		Gene	Stability	Gene	Stability value	Gene	SD [± Cq]	CV [%Cq]	Gene	Stability
‘Hakuho’	1	*EF-1α*	0.28	*ACTIN*	0.18	*HIS*	0.18	0.70	*ACTIN*	1.57
	2	*TUB-B*	0.28	*TUB-B*	0.20	*ACTIN*	0.31	1.31	*TUB-B*	1.86
	3	*ACTIN*	0.39	*EF-1α*	0.33	*TUB-B*	0.33	1.46	*EF-1α*	2.59
	4	*HIS*	0.46	*HIS*	0.40	*GAPDH*	0.37	1.45	*HIS*	2.83
	5	*GAPDH*	0.51	*GAPDH*	0.43	*EF-1α*	0.40	1.69	*GAPDH*	4.73
	6	*UBC*	0.56	*UBC*	0.59	*UBC*	0.50	2.13	*UBC*	6.00
	7	*eIF-4α*	0.61	*eIF-4α*	0.65	*eIF-4α*	0.58	2.37	*eIF-4α*	7.00
	8	*TBP*	0.67	*TBP*	0.79	*TBP*	0.61	2.36	*TBP*	8.00
	9	*TUB-A*	0.78	*TUB-A*	1.10	*TUB-A*	0.86	3.39	*TUB-A*	9.00
‘Xiacui’	1	*EF-1α*	0.20	*UBC*	0.11	*TUB-B*	0.17	0.76	*EF-1α*	1.41
	2	*UBC*	0.20	*EF-1α*	0.14	*EF-1α*	0.18	0.81	*UBC*	1.57
	3	*TUB-B*	0.28	*HIS*	0.27	*UBC*	0.23	0.98	*TUB-B*	2.45
	4	*HIS*	0.31	*TUB-B*	0.28	*HIS*	0.29	1.17	*HIS*	3.72
	5	*ACTIN*	0.34	*GAPDH*	0.28	*ACTIN*	0.30	1.28	*ACTIN*	5.23
	6	*GAPDH*	0.37	*ACTIN*	0.33	*TBP*	0.36	1.35	*GAPDH*	6.19
	7	*TBP*	0.40	*TBP*	0.40	*GAPDH*	0.38	1.58	*TBP*	6.48
	8	*TUB-A*	0.45	*TUB-A*	0.60	*TUB-A*	0.47	1.96	*TUB-A*	8.00
	9	*eIF-4α*	0.85	*eIF-4α*	2.21	*eIF-4α*	1.38	5.38	*eIF-4α*	9.00
‘Fantasia’	1	*UBC*	0.32	*UBC*	0.27	*GAPDH*	0.61	2.44	*UBC*	1.32
	2	*ACTIN*	0.32	*TUB-B*	0.30	*TUB-A*	0.72	2.85	*TUB-B*	2.78
	3	*TUB-B*	0.34	*TBP*	0.32	*UBC*	0.73	2.92	*GAPDH*	2.99
	4	*GAPDH*	0.37	*GAPDH*	0.35	*eIF-4α*	0.86	3.33	*ACTIN*	3.08
	5	*TBP*	0.41	*ACTIN*	0.35	*ACTIN*	0.88	3.66	*TBP*	4.53
	6	*eIF-4α*	0.47	*eIF-4α*	0.39	*TUB-B*	0.88	3.68	*eIF-4α*	5.42
	7	*EF-1α*	0.52	*EF-1α*	0.62	*TBP*	0.99	3.85	*TUB-A*	5.66
	8	*TUB-A*	0.58	*TUB-A*	0.64	*HIS*	1.01	3.78	*EF-1α*	7.45
	9	*HIS*	0.66	*HIS*	0.85	*EF-1α*	1.20	4.98	*HIS*	8.74
‘NJC108’	1	*UBC*	0.50	*UBC*	0.25	*HIS*	0.50	1.91	*UBC*	1.78
	2	*ACTIN*	0.50	*ACTIN*	0.25	*TBP*	0.59	2.24	*ACTIN*	1.93
	3	*TUB-B*	0.63	*TUB-B*	0.34	*GAPDH*	0.72	2.82	*HIS*	3.13
	4	*HIS*	0.75	*eIF-4α*	0.42	*TUB-A*	0.84	3.24	*TUB-B*	3.57
	5	*GAPDH*	0.79	*GAPDH*	0.71	*UBC*	0.97	3.96	*GAPDH*	4.4
	6	*eIF-4α*	0.84	*HIS*	0.78	*TUB-B*	1.10	4.51	*TBP*	5.66
	7	*TUB-A*	0.92	*TUB-A*	1.14	*ACTIN*	1.12	4.58	*eIF-4α*	5.83
	8	*TBP*	1.03	*TBP*	1.50	*eIF-4α*	1.24	5.01	*TUB-A*	6.09
	9	*EF-1α*	1.65	*EF-1α*	3.74	*EF-1α*	3.92	15.34	*EF-1α*	9.00

**Table 4:** The stability ranking of candidate reference genes in different stage of samples by geNorm, NormFinder, BestKeeper and RefFinder.

Stage	Rank	geNorm		NormFinder		BestKeeper			RefFinder	
		Gene	Stability	Gene	Stability value	Gene	SD [± Cq]	CV [%Cq]	Gene	Stability
S1	1	*TUB-B*	0.32	*TUB-A*	0.27	*TUB-A*	0.33	1.36	*TUB-A*	1.41
	2	*HIS*	0.32	*ACTIN*	0.38	*UBC*	0.36	1.47	*TUB-B*	2.38
	3	*UBC*	0.41	*EF-1α*	0.38	*eIF-4α*	0.44	1.76	*UBC*	3.50
	4	*TUB-A*	0.48	*TUB-B*	0.40	*TUB-B*	0.45	1.96	*ACTIN*	3.94
	5	*EF-1α*	0.54	*UBC*	0.41	*ACTIN*	0.48	2.01	*HIS*	4.14
	6	*ACTIN*	0.57	*eIF-4α*	0.50	*HIS*	0.48	1.92	*EF-1α*	4.21
	7	*eIF-4α*	0.59	*HIS*	0.60	*EF-1α*	0.51	2.23	*eIF-4α*	5.24
	8	*GAPDH*	0.64	*GAPDH*	0.70	*GAPDH*	0.70	2.81	*GAPDH*	8.00
	9	*TBP*	0.78	*TBP*	1.15	*TBP*	0.77	2.90	*TBP*	9.00
S2	1	*UBC*	0.54	*UBC*	0.25	*HIS*	0.64	2.49	*UBC*	1.78
	2	*HIS*	0.54	*TUB-B*	0.34	*GAPDH*	0.78	3.10	*HIS*	1.86
	3	*TUB-A*	0.59	*ACTIN*	0.34	*TBP*	0.88	3.39	*ACTIN*	2.91
	4	*ACTIN*	0.66	*HIS*	0.52	*TUB-A*	0.91	3.62	*TUB-A*	3.94
	5	*TUB-B*	0.71	*TUB-A*	0.56	*UBC*	0.99	4.03	*TUB-B*	4.33
	6	*GAPDH*	0.78	*GAPDH*	0.75	*ACTIN*	1.06	4.35	*GAPDH*	4.56
	7	*TBP*	0.97	*TBP*	1.50	*TUB-B*	1.43	6.08	*TBP*	5.66
	8	*eIF-4α*	1.33	*eIF-4α*	2.40	*eIF-4α*	1.70	6.50	*eIF-4α*	8.00
	9	*EF-1α*	1.88	*EF-1α*	3.68	*EF-1α*	3.91	15.33	*EF-1α*	9.00
S3	1	*GAPDH*	0.43	*EF-1α*	0.36	*ACTIN*	0.28	1.18	*ACTIN*	1.73
	2	*ACTIN*	0.43	*UBC*	0.38	*TBP*	0.36	1.38	*EF-1α*	1.86
	3	*EF-1α*	0.51	*ACTIN*	0.48	*GAPDH*	0.44	1.78	*GAPDH*	2.63
	4	*UBC*	0.60	*GAPDH*	0.49	*EF-1α*	0.55	2.41	*UBC*	2.99
	5	*TUB-A*	0.66	*TUB-B*	0.62	*UBC*	0.64	2.67	*TBP*	5.12
	6	*TUB-B*	0.68	*TUB-A*	0.63	*eIF-4α*	0.67	2.71	*TUB-B*	5.69
	7	*TBP*	0.75	*TBP*	0.80	*TUB-B*	0.78	3.35	*TUB-A*	6.16
	8	*eIF-4α*	0.82	*eIF-4α*	0.86	*TUB-A*	0.88	3.54	*eIF-4α*	7.44
	9	*HIS*	0.88	*HIS*	0.89	*HIS*	0.89	3.46	*HIS*	9.00
S4	1	*TUB-B*	0.44	*ACTIN*	0.23	*TUB-A*	0.70	2.67	*ACTIN*	1.73
	2	*HIS*	0.44	*TUB-B*	0.41	*GAPDH*	0.72	2.85	*TUB-B*	2.11
	3	*ACTIN*	0.65	*UBC*	0.52	*ACTIN*	0.78	3.22	*HIS*	3.25
	4	*UBC*	0.68	*HIS*	0.61	*TBP*	0.90	3.40	*UBC*	3.83
	5	*GAPDH*	0.73	*GAPDH*	0.62	*TUB-B*	0.96	4.04	*GAPDH*	3.98
	6	*EF-1α*	0.78	*EF-1α*	0.77	*UBC*	1.08	4.46	*TUB-A*	5.20
	7	*TBP*	0.86	*TBP*	0.82	*HIS*	1.11	4.32	*TBP*	6.09
	8	*eIF-4α*	0.93	*eIF-4α*	1.12	*EF-1α*	1.28	5.33	*EF-1α*	6.45
	9	*TUB-A*	1.04	*TUB-A*	1.30	*eIF-4α*	1.35	5.40	*eIF-4α*	8.24

### BestKeeper

The BestKeeper software evaluates the expression stability of reference genes with SD and CV of Cq values, and the smaller SD and CV value suggest the more stable results. For the three sets (Tables 2, 3, 4), the order of the stability of the reference genes in ‘Total’ is as follows: *ACTIN* > *GAPDH* > *UBC* > *TBP* > *HIS* > *TUB-B* > *TUB-A* > *eIF-4α* > *EF-1α*; the gene *HIS* ranks the first in ‘Hakuho’ and ‘NJC108’, but ranks the fourth in ‘Xiacui’ and the eighth in ‘Fantasia’; the gene *TUB-A* ranks the first in S1 and S4, but the seventh and eighth in S2 and S3, respectively. According to the analysis of BestKeeper, the genes *ACTIN* and *GAPDH* have good stability, while *EF-1α* has the worst stability.

### RefFinder

The stability ranking calculated by the three software geNorm, NormFinder and BestKeeper were not entirely consistent. RefFinder is used to integrate the results to get the comprehensive index ranking. As shown in Tables 2, 3 and 4, the genes *ACTIN* and *UBC* have good stability, while *eIF-4α* and *EF-1α* have poor stability in ‘Total’ set. The order of stability was: *ACTIN* > *UBC* > *GAPDH* > *TUB-B* > *HIS* > *TBP* > *TUB-A* > *eIF-4α* > *EF-1α*. The genes *ACTIN* and *EF-1α* rank first in ‘Hakuho’ and ‘Xiacui’, respectively. The gene *UBC* ranks first in ‘Fantasia’, ‘NJC108’, and ranks the second in ‘Xiacui’, while ranks the sixth in ‘Hakuho’. The gene *ACTIN* shows the highest stability in S2, S3 and S4, while ranks the fourth in S1.

### Analysis of the optimal number of reference genes of peach

**Figure 4:**
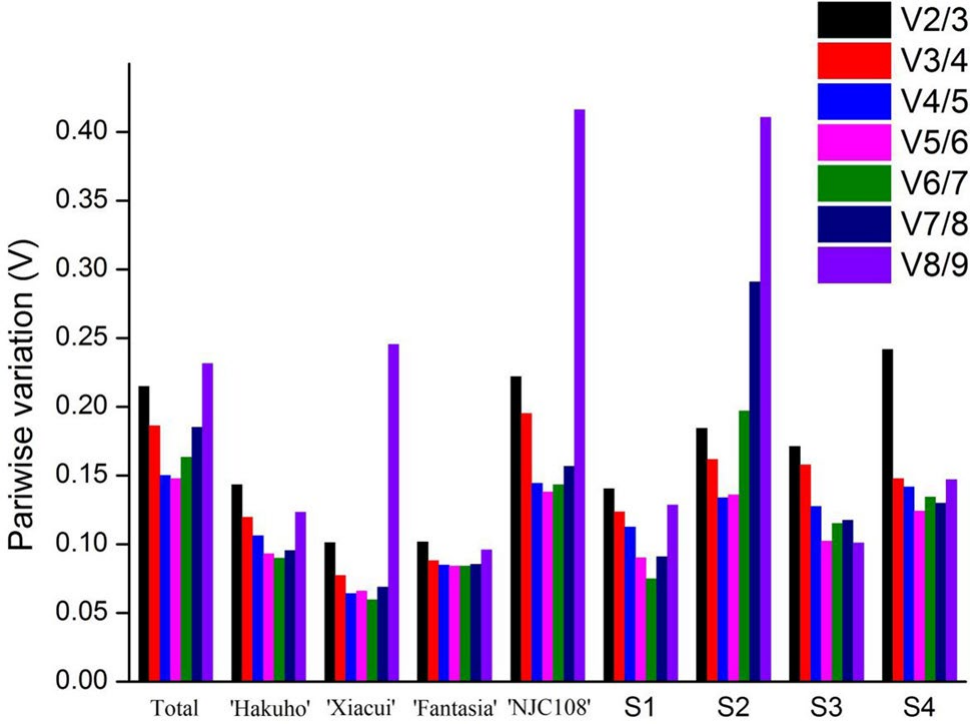
Pairwise variation analysis to determination of the optimal number of the nine candidate reference genes. All pairwise variation (Vn/Vn + 1) were calculated via geNorm, and the values determined the minimum number of reference genes for accurate normalization in each experimental set. The Vn/Vn + 1 values below 0.15 indicate that an additional reference genes are not necessary for gene expression normalization.

The geNorm software was used to determine the optimal number of reference genes by analyzing the pairwise variation (Vn/n + 1) between normalization factors (NFn and NFn + 1, n ≥ 2). When Vn/(n + 1) < 0.15, it indicates that an extra reference gene is not necessary, whereas Vn/(n + 1) ≥ 0.15 means that at least n + 1 genes should be required in qRT-PCR analysis. As shown in Fig. 4, in the ‘Total’ set, only the value of V5/6 is less than 0.15, indicating that five reference genes (*ACTIN*, *UBC*, *GAPDH*, *TUB-B* and *HIS*) were proposed to be used. For ‘Hakuho’ (*ACTIN* and *TUB-B*), ‘Xiacui’ (*TUB-B* and *EF-1α*), ‘Fantasia’ (*UBC* and *TUB-B*) and S1 (*TUB-A* and *TUB-B*), two reference genes were enough with the V2/3 less than 0.15, while four genes were needed in ‘NJC108’ (*UBC*, *ACTIN*, *HIS* and *TUB-B*), S2 (*UBC*, *HIS*, *ACTIN* and *TUB-A*) and S3 (*ACTIN*, *EF-1α*, *GAPDH* and *UBC*) with the V4/5 dropping to 0.15. For S4, three genes (*ACTIN*, *TUB-B* and *HIS*) needs to be introduced.

### Determination of flesh firmness and validation of the selected reference genes of peach

**Figure 5:**
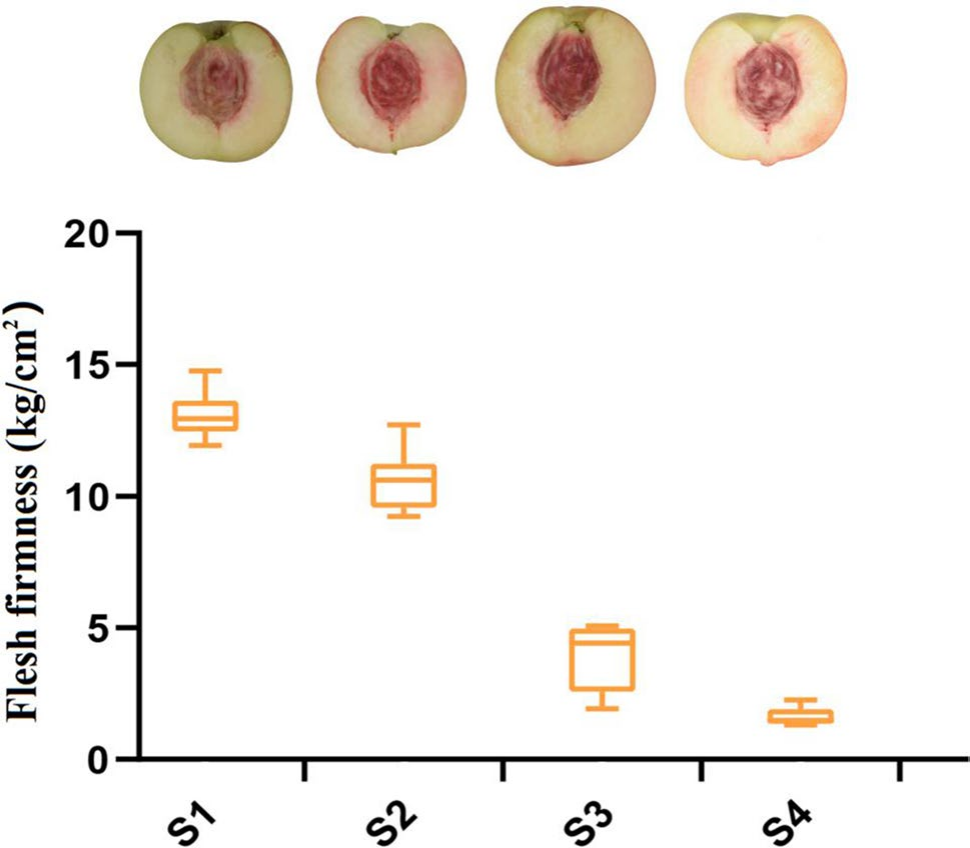
The fruit sampling stages and the flesh firmness of ‘Hakuho’ during fruit ripening and softening. The samples were randomly collected from trees at 4-day intervals for four times, starting from nearly 85 days after full bloom (DAFB) through to ripening, and designated as S1, S2, S3 and S4, respectively.

**Figure 6:**
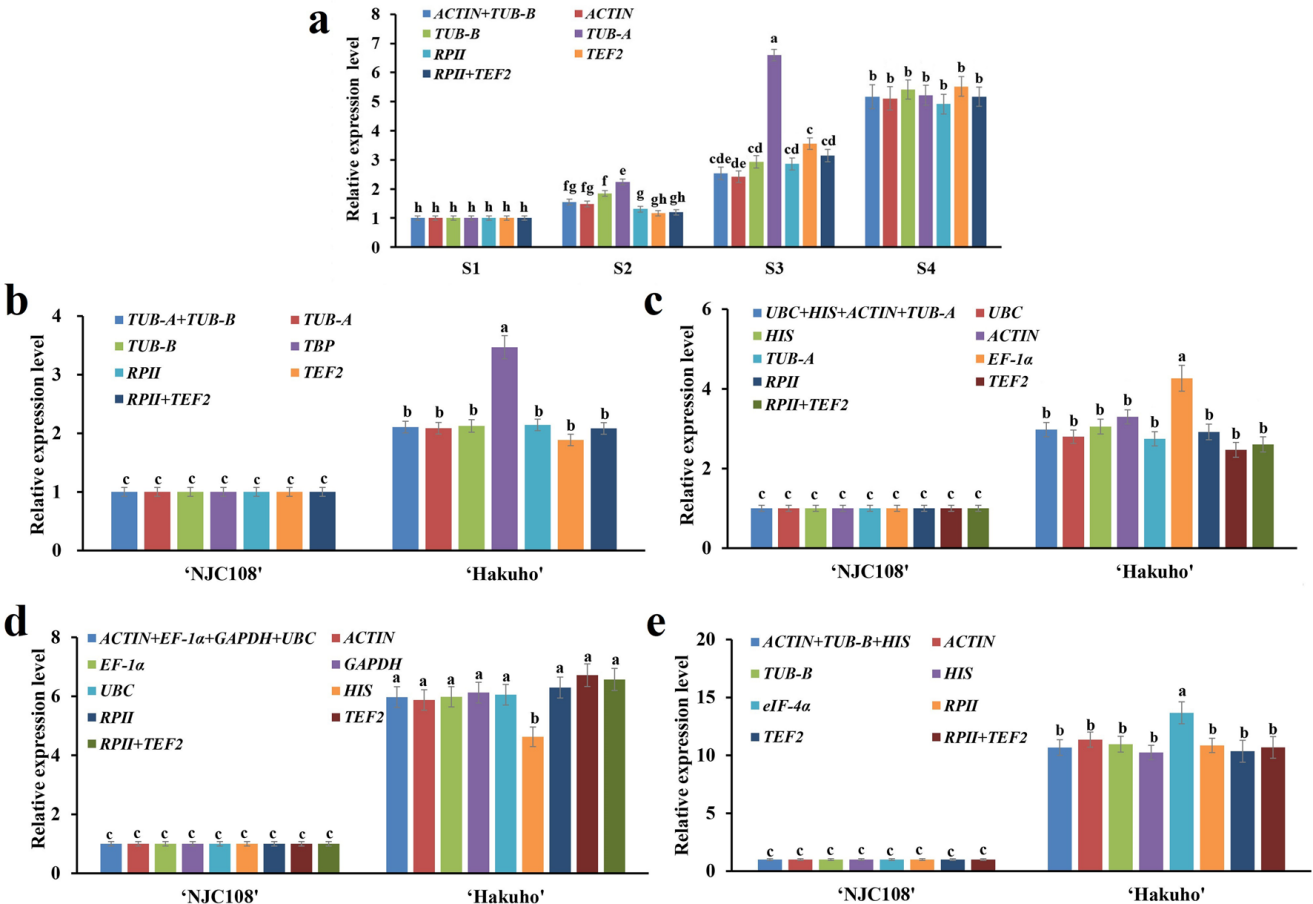
Relative expression level of *PpACS1* gene in ‘Hakuho’ peach cultivar from S1 to S4 stage. (**a**) Genes were normalized to *ACTIN*, *TUB-B*, *TUB-A*, *RPII*and *TEF2*, individually and in combination, in ‘Hakuho’ peach cultivar. (**b**) Genes were normalized to *TUB-A*, *TUB-B*, *TBP*, *RPII*and *TEF2*, individually and in combination, in S1. (**c**) Genes were normalized to *UBC*, *HIS*, *ACTIN*, *TUB-A*, *EF-1α*, *RPII* and *TEF2*, individually and in combination, in S2. (**d**) Genes were normalized to *ACTIN*, *EF-1α*, *GAPDH*, *UBC*, *HIS*, *RPII* and *TEF2*, individually and in combination, in S3. (**e**) Genes were normalized to *ACTIN*, *TUB-B, HIS*, *eIF-4α*, *RPII* and *TEF2*, individually and in combination, in S4. The statistical significance was determined by Duncan’s multiple comparison tests. Different letters indicate a significant difference (P < 0.05).

Verifying the expression stability of candidate reference genes in different sets will greatly improve the reliability of gene expression data. In this study, the sampling stages and the flesh firmness of ‘Hakuho’ during four fruit ripening stages were displayed (Fig. 5). The relative expression level of *PpACS1*, encoding 1-aminocyclopropane-1-carboxylic acid synthase in ‘Hakuho’ samples during fruit development were evaluated using the several top ranking reference genes, as recommended by RefFinder, alone or with a combination for data normalization. Combined with the optimal number of reference genes recommended by geNorm, the reference genes including the optimal reference genes of *ACTIN* and *TUB-B* in ‘Hakuho’, *TUB-A* and *TUB-B* in S1, *UBC*, *HIS*, *ACTIN* and *TUB-A* in S2, *ACTIN*, *EF-1α*, *GAPDH* and *UBC* in S3, *ACTIN*, *TUB-B* and *HIS* in S4 (combined Fig. 4, Tables 3, 4), two stable reference genes *PpRPII* (encoding RNA polymeraseII) and *PpTEF2* (encoding translation elongation factor 2) have been reported in previous study^27^, and the least stable reference genes (*TUB-A*, *TBP*, *EF-1α, HIS* and *eIF-4α*) from the sample set, respectively. As illustrated in Fig. 6a, the *PpACS*1 expression level showed the similar (but not identical) trend when using single or a combination of reference genes *ACTIN*, *TUB-B* and *RPII*, *TEF2*. The expression level of *PpACS*1 increased gradually from S1 to S3*,* and increased rapidly from S3 to S4. There is no significant difference among the expression level of *PpACS1* normalized by most stable genes *ACTIN* , *TUB-B*, and those normalized by control internal reference genes *RPII* and *TEF2*, individually and in combination, in S1 and S4 stages (Fig. 6a). However, *PpACS1* expression level normalized by *TUB-B* showed significant difference with the expression level of *PpACS1* when *RPII* and *TEF2*, individually and in combination, were used as internal control genes in S2. In addition, *PpACS1* expression level normalized by *ACTIN* showed significant difference with the expression level of *PpACS1* when *TEF2* was used as internal control genes in S3. *PpACS1* expression level when normalized with the least stable gene *TUB-A* showed significant difference with *PpACS1* expression level when *ACTIN*, *TUB-B* and *RPII TEF2,* individually and in combination, were used as internal control genes from S1 to S4 stage. Furthermore, alone or the combination of two (*TUB-A* and *TUB-B*) or two (*RPII* and *TEF2*) reference genes were used to normalize qRT-PCR data showed the same expression patterns of *PpACS1* in S1. However, using the least stable gene *TBP* as internal control, the expression level of *PpACS1* showed significant difference with the data when normalized using other optimal reference genes individually or in combination (Fig. 6b) and the results were in accordance with expression level of *PpACS1* when the least stable reference genes: *EF-1α*, *HIS* and *eIF-4α* were used for normalization in S2, S3 and S4 stage, respectively (Fig. 6c–e). Taken together, these results indicate that using inappropriate internal reference genes for normalization may lead to in accuracy qRT-PCR results. Therefore, select appropriate and reliable control genes is essential for gene expression analysis.

## Discussion

To date, high throughput sequencing^29^, microarray^30^ and qRT-PCR^31^ approaches have been applied to detect gene expression, qRT-PCR has become a powerful tool and the most frequently used approach for normalization because of its sensitivity and specificity^8^. Accurate results of qRT-PCR are closely related to the normalization of certain suitable reference gene, and using of inappropriate reference gene will lead to deviated analysis and even wrong conclusions^32–34^. A good reference gene should have consistent expression levels under any experimental conditions and be independent of organs, tissues, stages of development and various treatments, etc. However, none of the reference genes has a constant expression profile under all experimental conditions. Therefore, screening and evaluating the best stable reference genes as well as establishing an effective evaluation system of multiple reference genes in specific research models are important for improving the accuracy of qRT-PCR experiments and clarifying qRT-PCR results-represented scientific questions in those models.

According to the textural changes during ripening, peach fruits are mainly classified into three types, melting, non-melting and stony hard^35^. Although there have been some reports on the selection of reliable reference genes in peach based on geNorm, NormFinder and/or BestKeeper software, and *TEF2, UBQ10* and *RPII*^25–27^; *eIF-1A*, *MUB6*^25^ and *miR5059*, *miR5072*^26^ were found to be the most suitable reference genes across the special set of all samples. In this study evaluated the expression stabilities of 9 reference genes in 16 experimental samples (4 peach cultivars and 4 peach ripening and softening stages) were evaluated using qRT-PCR to determine the most stable reference genes. The present study is the first and systematical survey focused on the stability analysis of reference genes in the three types of peach fruit during its ripening and softening, geNorm, NormFinder, BestKeeper and RefFinder were applied.

Different rankings for the 9 candidate reference genes were developed after comparison to the ranking of these candidates generated by the four algorithms (Tables 2, 3, 4). Based on calculations by geNorm, 5 optimal reference genes were selected, and *UBC*, *ACTIN* expressed the most stably in the ‘Total’ sample, and they were considered to be the most suitable reference candidate genes under situations that different cultivars and ripening stages are blended. The NormFinder software showed nearly the same results in the ‘Total’ sample as geNorm. Based on the discrepancies of the statistical algorithms between the two software, geNorm and NormFinder, the reference gene stability ranking varied in the different cultivar and stage setups between these two programs (Tables 3, 4)^36–38^. The geNorm is highly dependent on the assumption that none of the genes is co-regulated as this would lead to an erroneous choice of optimum normalizer pair^25,27,37^, The geNorm provide more reliable results than NormFinder in terms of the best reference pairs for each experimental setup (Tables 2, 3, 4). In BestKeeper analysis, it is found that *ACTIN* and *GAPDH* had relatively good stability, *EF-1α* has the worst performance in the ‘Total’ setup. In view of the discrepancies among the analysis results of the above three software. RefFinder was further introduced to integrate all the results to avoid one sidedness of single software analysis and make the screening results more reliable. Comprehensive analysis by RefFinder software, the stability ranking turned out to be *ACTIN* > *UBC* > *GAPDH* > *TUB-B* > *HIS* > *TBP* > *TUB-A* > *eIF-4α* > *EF-1α*. Therefore, *ACTIN* and *UBC* are recommended as the most suitable reference genes, while *ACTIN*, *UBC*, *GAPDH*, *TUB-B* and *HIS* are the optimal reference genes when research model involves total analysis of different cultivars and ripening stages. In fact, the stability of the reference gene is not the same across plants. For example, *ACTIN,* the third mainly used reference gene, has been widely used as reference gene in gene expression studies in many organisms^27^, and *ACTIN* is stably expressed in this experiment, however *ACTIN* performed unsatisfactory in wheat^39^, maize^40^ and garlic^41^. This partly attributed to the fact that *ACTIN* as one of the major components of cytoplasmic microfilaments in eukaryotic cells, not only supports the cell and determines its shape but also participates in other cellular functions^42^. *GAPDH* has only moderate stability in the ‘Total’, ‘Fantasia’ and ‘S3’ experimental setups. And some researches have showed that *GAPDH* had a good performance in grape^43^ and *Primula forbesii*^12^, but not in wheat^39^. *EF-1 α* can be used as an appropriate reference gene in zucchini^44^, wolfberry^45^, and wheat^39^, but our analysis showed that *EF-1 α* was not the reliable gene for comparative expression in our peach model. *UBC* is a reference gene with good expression stability in all samples in different development stages of pearl millet^46^, which also behaves well in current peach model, but the expression stability is the worst in different tissues and fruit stages of pitaya fruit^47^.

Fruit ripening and softening is a complex process involving major traditions in fruit development and metabolism, and this developmental transition involves coordinated changes in a number of biochemical pathways. Ethylene regulates at least part of this developmental transition, ethylene biosynthesis, ethylene responses, and ethylene-regulated gene expression have been extensively studied in ripening fruit^18^. *ACS* is a key rate-limiting enzyme that controls ethylene synthesis in plants and plays an important regulatory role in plant development, fruit maturation^48,49^. The expressions of *ACS* homologous genes are differentially regulated by plant developmental and hormonal signals^50^ and *PpACS* gene expression is closely related to fruit ripening in peach^23^. Six *PpACS* genes (*PpACS1-6*) have been identified in nectarine (*Prunus persica* var. *nectarina*) and the expression level of the three genes (*PpACS1*, *PpACS4* and *PpACS5*) showed dynamically changed during fruit ripening and softening in peach^23^. Peach, a climatic fruit, undergoes textural changes that lead to loss of tissue firmness during fruit ripening and softening and is accompanied by an increase in ethylene evolution. The expression level of *PpACS1* which induces peach ripening, is related to ethylene production^51,52^.

To test the reliablity of the reference genes, the expression profiles of *PpACS1* were assayed in ‘Hakuho’. Usually, only one reference gene was used for qRT-PCR data normalization in most reported gene expression studies^53^. However, single reference gene is insufficient sometimes^54^. RefFinder, which considers the optimal reference gene results of three algorithms (geNorm, NormFinder and BestKeeper) together. Therefore, *PpACS1* expression level of ‘Hakuho’ during S1 to S4 were analyzed using several most stable reference genes recommended by RefFinder and the stable genes (*TEF2* and *RP II*) reported in previous study^27^ individually and in combination to confirm the importance of selecting appropriate reference genes in experimental design. Combined the firmness of ‘Hakuho’ during peach ripening and softening and the expression level of *PpACS1* which was normalized by *ACTIN*, *TUB-B, TUB-A, RPII*and *TEF2* genes alone or a combination, the firmness of ‘Hakuho’ was decreased from 13.67 kg/cm^2^ to 1.61 kg/cm^2^ during fruit ripening and softening stage (from S1 to S4) (Fig. 5). The *PpACS1* expression level when normalized by *RPII and TEF2* in S4 was 3.9 and 4.5 times higher than that in S1, respectively, while the expression level of *PpACS1* which was normalized by the most stable reference genes *ACTIN* and *TUB-B* in S4 was 4.1 and 4.4 times higher than that in S1. In addition, we found that *PpACS1* expression level normalized by *ACTIN* showed no significant difference with the expression level of *PpACS1* normalized by *RPII and TEF2* from S1 to S4 (Fig. 6a). And this results just confirmed that *ACTIN* was the best stable reference gene in ‘Hakuho’ (Table 4). *PpACS1* expression showed the same trend from S1 to S4 when *ACTIN*, *TUB-B, RPII* and *TEF2* were used as internal genes. While the most unstable gene *TUB-A* was used for normalization, *PpACS1* expression level was severely overestimated during S3 stage in ‘Hakuho’ (Fig. 6a).

Generally speaking, flesh texture types of peach including the non-melting type and the stony hard type in addition to the melting type^55^. Mature peach fruit of melting and non-melting flesh types produce ethylene during ripening and this character is controlled by the related genes such as *PpACS1*, however, stony hard fruit texture is characterized by the absence of both ethylene production and post-harvest softening in mature fruit^56^. The melting type is characterized by rapid softening of the fruit flesh, while the non-melting type is characterized by a more limited softening. To validate the candidate reference genes at different ripening stages (S1: *TUB-A* and *TUB-B*, S2: *UBC*, *HIS*, *ACTIN* and *TUB-A*, S3: *ACTIN*, *EF-1α*, *GAPDH* and *UBC*, S4: *ACTIN*, *TUB-B* and *HIS*) of ‘Hakuho’, these multiple reference genes were used as internal controls, individually and in combination, to normalize the expression level of *PpACS1*, while the non-melting peach cultivar ‘NJC108’ was used as a control (Fig. 6b–e). The results with the optimal reference genes alone or a combination as internal controls were in consistent with the reduction of the peach fruit firmness and the expression characteristics of *PpACS1* from S1 to S4 stage in ‘Hakuho’ (Figs. 5, 6a), while the *PpACS1* expression level with the least stable reference genes (S1: *TBP*, S2: *EF-1α*, S3: *HIS* and S4: *eIF-4α*) (Fig. 6b–e) showed discrepancy with the expression trend of *PpACS1* from S1 to S4 stage in ‘Hakuho’ (Fig. 6a). All the results fully showed that different reference genes were used to normalize in qRT-PCR analysis may lead different conclusions. Especially using an unstable reference gene, the results may be biased.

In conclusion, the selection of a suitable internal reference gene is a key step in gene expression analysis. The results from this study not only provide a favorable basis for selection of suitable reference genes in different cultivars and fruit maturation of peach, but also can be used as a reference for the exploration of related functional genes, expression patterns and regulatory mechanism in peach.

## Materials and methods

### Plant materials and growth conditions

Samples were collected from 8-year-old peach trees of ‘Hakuho’ (a melting flesh peach genotype), ‘NJC108’ (a non-melting flesh peach genotype), ‘Xiacui’ and ‘Fantasia’ (a stony hard flesh peach genotype) cultivar. All these four peach cultivars were growing in the National Peach Germplasm Repository of Zhengzhou Fruit Research Institute (Henan, China) under the same common field conditions. The collection of these peach cultivars was permitted by Zhengzhou Fruit Research Institute and it complies with local and national guidelines and legislation. Peach fruits with no visible defects, at a stage equivalent to commercial ripeness, were randomly collected from trees at 4-day intervals for four times, starting from nearly 85 days after full bloom (DAFB) through to ripening and softening, and designated as S1, S2, S3 and S4, respectively. Samples were collected and were immediately frozen in liquid nitrogen and then stored at − 80 °C until RNA extraction. Three biological replicates were performed for each sample.

### Total RNA isolation and cDNA synthesis

Total RNA of all samples were extracted by using an RNA kit (Tiangen, Beijing, China) in accordance with the manufacturer’s method. The quality and purity of total RNA were further assessed with spectrophotometer NanoDrop 2000C (Thermo, USA) and 1% agarose gel electrophoresis. Approximately 1000 ng of total RNA was reverse transcribed into cDNA using the Prime Script RT Reagent Kit (TransGene, Beijing, China). The cDNA was subjected to tenfold serial dilutions (10×, 10^2^×, 10^3^×, 10^4^×, 10^5^×) for determining the amplification efficiency (E) and coefficient of correlation (*R*^2^) analysis; and 20-fold diluted for PCR amplification.

### Gene selection, primer design and gene cloning

Nine candidate reference genes, *EF-1α*, *GAPDH*, *TBP*, *UBC*, *eIF-4α*, *TUB-A*, *TUB-B*, *ACTIN* and *HIS*, were selected from the whole-genome of peach. Primer Premier 6 was used to design the specific primers according to the gene sequences. The nine candidate reference genes were cloned from peach with the primer sequences listed in Table S1.

### qRT-PCR, and statistical analysis of gene expression stability

The qRT-PCR was performed with a Bio-Rad real-time PCR System (Bio-Rad, CA, USA), with a SYBR qRT-PCR Mix (TsingKe, Beijing, China). The amplification procedure was conducted as follows: 95 °C for 1 min pre-denaturation, followed by 40 cycles at 95 °C for 5 s for denaturation, 60 °C for 30 s for annealing and extension, and melting curve analysis (61 cycles) at 65 °C for 10 s. The primer sequences of nine reference genes for qRT-PCR were listed in Table 1. Each assay contained a standard curve based on different dilutions of cDNA template to test the amplification efficiency (E, E% = (− 1 + 10 ^[−1/slope]^) × 100%) of primer pair of each gene. Controls without template were also included in each run.

Expression levels of the nine candidate reference genes of peach were quantified by the number of amplification cycles (Cq). Four software, geNorm^57^, NormFinder^58^, BestKeeper^59^, and RefFinder^60^ were used to evaluate the gene expression stability for all samples. For geNorm and NormFinder software, Cq values would be converted into relative quantities according to the formula 2^−ΔCq^, ΔCq = the corresponding Cq—minimum Cq. geNorm evaluates the stability of the reference gene expression by comparing the pairwise variation (M). Besides, the optimal number of reference genes in qRT-PCR normalization were determined by the pairwise variation (Vn/n + 1) among normalization factors (NFn and NFn + 1, n ≥ 2) in geNorm. NormFinder compares the intra- and inter-group variation and combines the variation into a stability value for each gene. BestKeeper could analyze the raw Cq value to rank the stability with standard deviation (SD) and coefficient of variation (CV). RefFinder calculates a comprehensive stability ranking by integrating the three computational programs (geNorm, Normfinder, and BestKeeper).

### Determination of flesh firmness and validation of reference genes of peach

Peach flesh firmness was determined with a fruit pressure tester (GY-4-J; TOP, China) equipped with an 11-mm diameter probe. The probe was pressed into the tissue of peach surface to 10 mm depth in every single smooth motion, and three disks were removed from opposite sides of each peach. Three peaches per sample were measured.

*ACS1*, a gene encoding an ethylene synthase and involved into fruit maturation, was selected to validate the suitable reference genes. The primer sequence of *PpACS1* was 5′-TGCGTGGAGCCTGGTTGGTT-3′ for forward and 5′-CGAACGAGAGGAGAGTGAGGAGAC-3′ for reverse. The optimal reference genes of *ACTIN* and *TUB-B* in ‘Hakuho’, two stable genes (*RPII*, *TEF2*) reported in previous study^27^, the least stable gene (*TUB-A*) and multiple reference gene combinations at different ripening stages in peach: S1 (*TUB-A* and *TUB-B*), S2 (*UBC*, *HIS*, *ACTIN* and *TUB-A*), S3 (*ACTIN*, *EF-1α*, *GAPDH* and *UBC*), S4 (*ACTIN*, *TUB-B* and *HIS*) were used as an internal standard to normalize *PpACS1* expression, respectively. The relative expression level of *PpACS1* gene in ‘Hakuho’ was calculated using 2^−ΔΔCt^ method^59^.

## Supplementary Information

Supplementary Information.
