# Supplementary material for: Selection and validation reference genes for qRT-PCR normalization in different cultivars during fruit ripening and softening of peach (Prunus persica)
Source: Sci Rep. 2021 Mar 31;11:7302. doi: 10.1038/s41598-021-86755-5 (PMC8012606; doi:10.1038/s41598-021-86755-5)
Supplement: Supplementary file 1 — Supplementary Information. [file 41598_2021_86755_MOESM1_ESM.docx]

**Selection and validation reference genes for qRT-PCR normalization in different cultivars during fruit ripening and softening of peach (*Prunus persica*)**

**Shuanghong You^1^†, Ke Cao^2^†, Changwen Chen^2^, Yong Li^2^, Jinlong Wu^2^, Gengrui Zhu^2^, Weichao Fang^2^, Xinwei Wang^2^, Lirong Wang****^2^*.**

**Address:**

^1^ Fruit Research Institute, Chongqing Academy of Agricultural Sciences, Chongqing 401329, China.

^2^ The Key Laboratory of Biology and Genetic Improvement of Horticultural Crops (Fruit Tree Breeding Technology), Ministry of Agriculture, Zhengzhou Fruit Research Institute, Chinese Academy of Agricultural Sciences, Zhengzhou 450009, China.

†These authors have contributed equally to this work.

***Correspondence:**

Lirong Wang

1. **mail:** [**wanglirong@caas.cn**](mailto:wanglirong@caas.cn)

**Supplementary information**


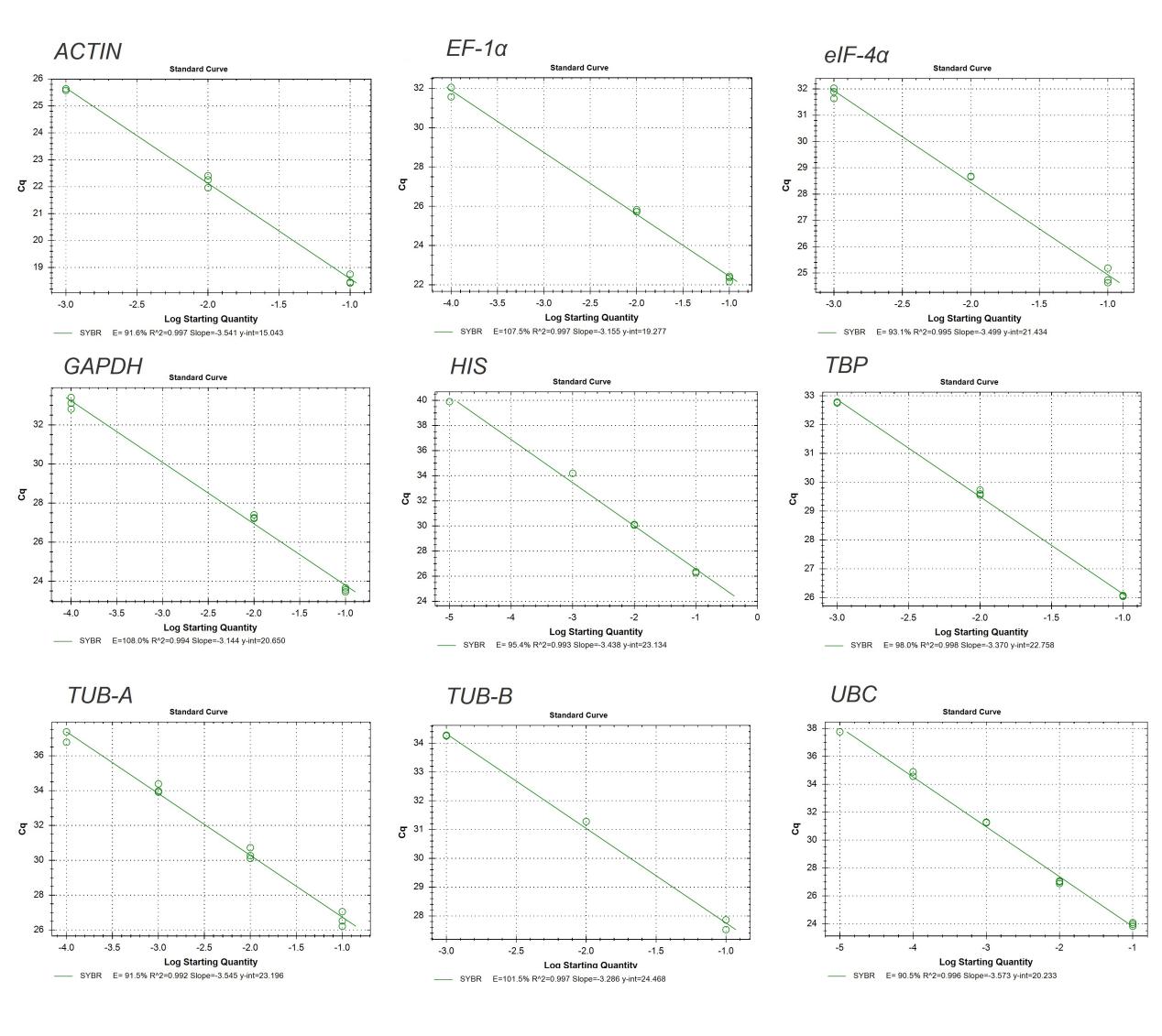


**Figure S1.** Standard curves of each candidate genes in peach.

**Table S1** Sequences primers and gene ID of nine genes used in this study

| **Gene symbol** | **Gene ID** | **Primer sequence (5’-3’)** |
| --- | --- | --- |
| *ACTIN* | Prupe.5G137100 | ATGGCAGATACCGAGGACATTC/  CTAGAAGCACTTTCTGTGAACGA |
| *EF-1a* | Prupe.6G095200 | ATGGGTAAAGAGAAGTTTCACATC/  TCACTTCTTCTTTGCTGCAGC |
| *GAPDH* | Prupe.5G155800 | GGAAGATCAAGATCGGAATCAAC/  CTAAGCATGATCAACAGATGCA |
| *TBP* | Prupe.6G001900 | ATGGCAGAACAAGGGTTAGAAG/  TCATTGCTGATTTTTCCTGAACT |
| *eIF-4α* | Prupe.3G045500 | AGGTGTTGCACCCGAAGGAT/  TCAGAGCAGATCAGCGACATT |
| *TUB-A* | Prupe.6G212700 | ATGAGGGAGTGCATCTCCATTC/  TCAGTAGTCGTCTCCCTCATC |
| *TUB-B* | Prupe.1G364800 | ATGAGAGAGATCTTGCATATCCAA/  TTAGTCCTCATAGTTCTCTTCAGC |
| *UBC* | Prupe.4G167300 | ATGGCCAACAGTAACCTTCC/  TCACACACCACTTGCATATAAAC |
| *HIS* | Prupe.1G369600 | ATGGCTCGTACCAAACAGACT/  TTAAGCACGTTCGCCACG |

**Table S2** The Cq values of the nine candidate reference genes across all samples in qRT-PCR analysis

| **Variety** | **Stage** | | **replicate** | | | ***EF-1α*** | | ***GAPDH*** | | ***TBP*** | | ***UBC*** | ***eIF-4α*** | ***TUB-A*** | ***TUB-B*** | | ***ACTIN*** | | ***HIS*** | |
| --- | --- | --- | --- | --- | --- | --- | --- | --- | --- | --- | --- | --- | --- | --- | --- | --- | --- | --- | --- | --- |
| **‘Hakuho’** | | **S1** | | **1-1** | 24.16 | | 25.97 | | 26.10 | | 24.80 | | 25.11 | 24.81 | 23.23 | 24.89 | | 25.20 | |  |
|  |  |  |  | **1-1** | 24.16 | | 25.96 | | 26.10 | | 24.57 | | 24.52 | 24.78 | 23.24 | 24.89 | | 25.15 | |  |
|  |  |  |  | **1-1** | 24.68 | | 25.76 | | 26.38 | | 25.21 | | 24.53 | 25.33 | 23.27 | 24.95 | | 25.14 | |  |
|  |  |  |  | **1-2** | 23.83 | | 25.67 | | 25.92 | | 23.66 | | 25.82 | 24.76 | 23.15 | 24.39 | | 25.12 | |  |
|  |  |  |  | **1-2** | 23.57 | | 25.56 | | 25.77 | | 23.68 | | 25.83 | 24.62 | 23.00 | 24.35 | | 25.09 | |  |
|  |  |  |  | **1-2** | 23.57 | | 25.78 | | 25.79 | | 24.06 | | 25.72 | 24.64 | 22.85 | 24.27 | | 25.21 | |  |
|  |  |  |  | **1-3** | 23.52 | | 27.13 | | 27.35 | | 23.90 | | 25.28 | 24.97 | 22.99 | 24.34 | | 24.96 | |  |
|  |  |  |  | **1-3** | 23.54 | | 27.24 | | 27.07 | | 23.72 | | 24.54 | 24.75 | 22.82 | 24.43 | | 24.87 | |  |
|  |  |  |  | **1-3** | 23.76 | | 27.21 | | 27.96 | | 24.00 | | 25.34 | 24.73 | 22.83 | 24.49 | | 25.00 | |  |
|  |  | **S2** | | **2-1** | 23.23 | | 25.38 | | 26.09 | | 23.71 | | 23.87 | 24.90 | 22.54 | 24.29 | | 25.17 | |  |
|  |  |  |  | **2-1** | 23.35 | | 25.62 | | 25.93 | | 23.60 | | 23.64 | 24.82 | 22.64 | 24.39 | | 25.17 | |  |
|  |  |  |  | **2-1** | 23.20 | | 25.40 | | 25.86 | | 23.80 | | 23.83 | 25.02 | 22.84 | 24.25 | | 25.14 | |  |
|  |  |  |  | **2-2** | 23.42 | | 25.32 | | 25.74 | | 23.78 | | 24.87 | 24.61 | 22.56 | 24.21 | | 25.29 | |  |
|  |  |  |  | **2-2** | 23.42 | | 25.50 | | 25.48 | | 23.87 | | 24.68 | 24.57 | 22.70 | 24.19 | | 25.22 | |  |
|  |  |  |  | **2-2** | 23.07 | | 25.43 | | 25.69 | | 25.53 | | 25.04 | 24.64 | 22.73 | 24.16 | | 25.44 | |  |
|  |  |  |  | **2-3** | 23.51 | | 25.55 | | 26.79 | | 23.18 | | 24.49 | 24.57 | 22.40 | 24.07 | | 25.09 | |  |
|  |  |  |  | **2-3** | 23.45 | | 25.53 | | 27.02 | | 23.19 | | 24.56 | 24.52 | 22.47 | 24.10 | | 25.12 | |  |
|  |  |  |  | **2-3** | 23.47 | | 25.42 | | 27.26 | | 23.40 | | 24.54 | 24.64 | 22.45 | 24.09 | | 25.14 | |  |
|  |  | **S3** | | **3-1** | 22.79 | | 25.14 | | 25.34 | | 23.33 | | 23.10 | 24.82 | 22.34 | 23.65 | | 25.02 | |  |
|  |  |  |  | **3-1** | 22.78 | | 25.08 | | 25.55 | | 23.27 | | 23.12 | 24.55 | 22.43 | 23.60 | | 25.01 | |  |
|  |  |  |  | **3-1** | 23.08 | | 25.08 | | 25.44 | | 23.92 | | 23.11 | 24.58 | 22.24 | 23.57 | | 25.02 | |  |
|  |  |  |  | **3-2** | 23.12 | | 25.19 | | 25.22 | | 22.70 | | 24.13 | 24.62 | 22.46 | 23.59 | | 25.18 | |  |
|  |  |  |  | **3-2** | 23.11 | | 25.19 | | 25.24 | | 23.00 | | 24.17 | 24.56 | 22.35 | 23.68 | | 25.57 | |  |
|  |  |  |  | **3-2** | 23.36 | | 25.12 | | 25.33 | | 23.37 | | 24.04 | 24.51 | 22.40 | 23.84 | | 25.38 | |  |
|  |  |  |  | **3-3** | 22.66 | | 25.09 | | 26.49 | | 22.53 | | 24.16 | 24.69 | 22.46 | 23.60 | | 24.94 | |  |
|  |  |  |  | **3-3** | 22.86 | | 25.28 | | 26.32 | | 22.73 | | 24.20 | 24.52 | 22.39 | 23.73 | | 25.06 | |  |
|  |  |  |  | **3-3** | 22.89 | | 25.30 | | 27.99 | | 23.10 | | 24.07 | 24.59 | 22.56 | 23.56 | | 25.36 | |  |
|  |  | **S4** | | **4-1** | 23.94 | | 24.75 | | 25.27 | | 23.40 | | 23.20 | 27.01 | 23.18 | 23.71 | | 24.78 | |  |
|  |  |  |  | **4-1** | 24.53 | | 24.99 | | 25.22 | | 23.41 | | 23.30 | 27.05 | 23.20 | 23.71 | | 24.76 | |  |
|  |  |  |  | **4-1** | 24.17 | | 25.03 | | 25.40 | | 23.48 | | 23.28 | 27.21 | 23.27 | 23.80 | | 24.84 | |  |
|  |  |  |  | **4-2** | 23.87 | | 25.35 | | 25.09 | | 24.13 | | 24.05 | 26.87 | 23.27 | 23.97 | | 24.63 | |  |
|  |  |  |  | **4-2** | 23.86 | | 25.38 | | 25.13 | | 24.08 | | 24.13 | 26.91 | 23.34 | 24.00 | | 24.57 | |  |
|  |  |  |  | **4-2** | 23.77 | | 25.34 | | 25.23 | | 23.92 | | 24.53 | 27.08 | 23.42 | 24.04 | | 24.72 | |  |
|  |  |  |  | **4-3** | 23.92 | | 25.32 | | 26.19 | | 22.69 | | 24.16 | 26.93 | 23.23 | 23.79 | | 24.87 | |  |
|  |  |  |  | **4-3** | 24.14 | | 25.46 | | 26.22 | | 22.65 | | 24.05 | 26.83 | 23.21 | 23.74 | | 24.78 | |  |
|  |  |  |  | **4-3** | 24.05 | | 25.42 | | 26.25 | | 23.21 | | 24.35 | 26.99 | 23.44 | 23.85 | | 24.92 | |  |
| **‘Xiacui’** | | **S1** | | **1-1** | 22.30 | | 24.32 | | 27.18 | | 23.94 | | 24.68 | 24.42 | 22.42 | 23.30 | | 24.47 | |  |
|  |  |  |  | **1-1** | 22.27 | | 24.32 | | 27.12 | | 23.71 | | 24.78 | 24.24 | 22.27 | 23.41 | | 24.40 | |  |
|  |  |  |  | **1-1** | 22.21 | | 24.31 | | 27.15 | | 23.91 | | 25.11 | 24.34 | 22.29 | 23.16 | | 24.19 | |  |
|  |  |  |  | **1-2** | 22.26 | | 24.16 | | 27.21 | | 23.89 | | 24.72 | 24.13 | 22.06 | 23.29 | | 24.29 | |  |
|  |  |  |  | **1-2** | 22.13 | | 24.00 | | 27.16 | | 23.99 | | 24.56 | 24.05 | 22.16 | 23.00 | | 24.31 | |  |
|  |  |  |  | **1-2** | 22.22 | | 23.81 | | 27.07 | | 23.93 | | 24.67 | 23.93 | 22.17 | 23.06 | | 24.32 | |  |
|  |  |  |  | **1-3** | 22.38 | | 24.17 | | 27.61 | | 23.90 | | 24.64 | 24.21 | 21.98 | 23.84 | | 24.54 | |  |
|  |  |  |  | **1-3** | 22.36 | | 24.17 | | 26.89 | | 23.72 | | 24.58 | 23.93 | 22.08 | 23.73 | | 24.23 | |  |
|  |  |  |  | **1-3** | 22.38 | | 24.13 | | 27.11 | | 23.70 | | 24.60 | 23.85 | 22.22 | 24.03 | | 24.48 | |  |
|  |  | **S2** | | **2-1** | 22.38 | | 24.76 | | 26.71 | | 23.99 | | 33.18 | 23.62 | 22.57 | 23.53 | | 25.12 | |  |
|  |  |  |  | **2-1** | 22.33 | | 24.80 | | 26.83 | | 23.87 | | 32.74 | 24.17 | 22.42 | 23.79 | | 25.01 | |  |
|  |  |  |  | **2-1** | 22.65 | | 25.10 | | 27.14 | | 24.18 | | 33.01 | 23.86 | 22.69 | 23.96 | | 25.32 | |  |
|  |  |  |  | **2-2** | 22.51 | | 24.76 | | 26.94 | | 23.95 | | 25.15 | 23.54 | 22.30 | 23.48 | | 25.23 | |  |
|  |  |  |  | **2-2** | 22.45 | | 24.58 | | 27.03 | | 24.01 | | 25.26 | 23.63 | 22.13 | 23.58 | | 25.36 | |  |
|  |  |  |  | **2-2** | 22.66 | | 24.78 | | 27.13 | | 24.16 | | 25.25 | 23.71 | 22.61 | 23.65 | | 25.17 | |  |
|  |  |  |  | **2-3** | 22.42 | | 25.17 | | 26.50 | | 24.02 | | 26.02 | 23.62 | 22.11 | 24.05 | | 24.88 | |  |
|  |  |  |  | **2-3** | 22.32 | | 25.10 | | 26.62 | | 24.10 | | 25.93 | 23.75 | 22.13 | 24.17 | | 24.91 | |  |
|  |  |  |  | **2-3** | 22.53 | | 25.17 | | 26.71 | | 24.10 | | 25.95 | 23.74 | 22.46 | 24.16 | | 24.95 | |  |
|  |  | **S3** | | **3-1** | 22.06 | | 23.98 | | 26.28 | | 23.56 | | 24.82 | 23.42 | 22.47 | 23.17 | | 24.46 | |  |
|  |  |  |  | **3-1** | 22.05 | | 23.89 | | 26.26 | | 23.57 | | 24.85 | 23.49 | 22.40 | 22.99 | | 24.61 | |  |
|  |  |  |  | **3-1** | 21.97 | | 24.11 | | 26.01 | | 23.37 | | 24.98 | 23.36 | 22.48 | 23.04 | | 24.98 | |  |
|  |  |  |  | **3-2** | 21.88 | | 24.00 | | 26.39 | | 23.21 | | 24.69 | 23.42 | 22.38 | 23.17 | | 24.25 | |  |
|  |  |  |  | **3-2** | 21.89 | | 23.91 | | 26.31 | | 23.25 | | 24.59 | 23.28 | 22.36 | 23.20 | | 24.41 | |  |
|  |  |  |  | **3-2** | 21.78 | | 24.13 | | 26.40 | | 23.20 | | 24.69 | 23.56 | 22.21 | 23.21 | | 24.33 | |  |
|  |  |  |  | **3-3** | 22.08 | | 23.86 | | 26.00 | | 23.44 | | 24.53 | 23.38 | 22.25 | 23.57 | | 24.60 | |  |
|  |  |  |  | **3-3** | 21.99 | | 23.75 | | 25.81 | | 23.33 | | 24.40 | 23.31 | 22.28 | 23.45 | | 24.62 | |  |
|  |  |  |  | **3-3** | 22.12 | | 23.60 | | 25.96 | | 23.38 | | 24.43 | 23.44 | 22.17 | 23.84 | | 25.27 | |  |
|  |  | **S4** | | **4-1** | 22.23 | | 24.11 | | 26.63 | | 23.71 | | 26.22 | 24.64 | 22.55 | 23.47 | | 24.58 | |  |
|  |  |  |  | **4-1** | 22.11 | | 24.19 | | 26.96 | | 23.88 | | 26.15 | 24.63 | 22.40 | 23.48 | | 24.30 | |  |
|  |  |  |  | **4-1** | 22.42 | | 24.41 | | 27.03 | | 23.86 | | 26.85 | 24.95 | 23.25 | 23.42 | | 24.54 | |  |
|  |  |  |  | **4-2** | 22.18 | | 23.73 | | 27.14 | | 23.77 | | 24.64 | 24.82 | 22.32 | 23.38 | | 24.61 | |  |
|  |  |  |  | **4-2** | 22.17 | | 23.66 | | 27.17 | | 23.67 | | 24.74 | 24.96 | 22.52 | 23.33 | | 24.74 | |  |
|  |  |  |  | **4-2** | 22.47 | | 23.43 | | 26.91 | | 23.61 | | 24.88 | 24.77 | 22.68 | 23.65 | | 24.83 | |  |
|  |  |  |  | **4-3** | 22.46 | | 23.54 | | 26.60 | | 23.54 | | 24.62 | 24.69 | 22.35 | 23.92 | | 24.51 | |  |
|  |  |  |  | **4-3** | 22.57 | | 23.78 | | 26.52 | | 23.59 | | 24.87 | 24.61 | 22.43 | 24.07 | | 24.44 | |  |
|  |  |  |  | **4-3** | 22.48 | | 23.76 | | 26.60 | | 23.54 | | 24.88 | 25.13 | 22.49 | 24.17 | | 24.53 | |  |
| **‘Fantasia’** | | **S1** | | **1-1** | 23.11 | | 24.92 | | 25.05 | | 24.38 | | 24.74 | 24.88 | 23.60 | 23.44 | | 25.79 | |  |
|  |  |  |  | **1-1** | 23.00 | | 25.02 | | 24.85 | | 24.33 | | 24.94 | 24.65 | 23.29 | 23.28 | | 26.26 | |  |
|  |  |  |  | **1-1** | 23.65 | | 24.82 | | 25.16 | | 24.46 | | 25.61 | 24.64 | 23.28 | 23.44 | | 25.86 | |  |
|  |  |  |  | **1-2** | 22.76 | | 25.04 | | 25.10 | | 25.29 | | 25.67 | 24.32 | 23.76 | 23.78 | | 25.59 | |  |
|  |  |  |  | **1-2** | 22.62 | | 24.76 | | 24.87 | | 24.84 | | 25.58 | 24.32 | 23.78 | 23.54 | | 25.83 | |  |
|  |  |  |  | **1-2** | 22.89 | | 24.64 | | 25.12 | | 25.00 | | 26.34 | 24.88 | 23.91 | 23.62 | | 26.36 | |  |
|  |  |  |  | **1-3** | 22.88 | | 25.12 | | 25.38 | | 24.37 | | 24.87 | 24.55 | 23.37 | 23.86 | | 26.05 | |  |
|  |  |  |  | **1-3** | 22.74 | | 25.10 | | 25.24 | | 24.27 | | 24.82 | 24.47 | 23.37 | 23.40 | | 26.11 | |  |
|  |  |  |  | **1-3** | 22.91 | | 25.01 | | 25.52 | | 24.62 | | 25.22 | 24.53 | 23.67 | 23.41 | | 26.20 | |  |
|  |  | **S2** | | **2-1** | 23.00 | | 23.97 | | 24.37 | | 24.27 | | 24.78 | 24.79 | 22.73 | 23.11 | | 25.49 | |  |
|  |  |  |  | **2-1** | 23.07 | | 24.05 | | 24.73 | | 24.34 | | 24.39 | 24.74 | 22.71 | 23.01 | | 25.25 | |  |
|  |  |  |  | **2-1** | 23.33 | | 24.16 | | 24.55 | | 24.17 | | 24.74 | 24.90 | 23.09 | 23.11 | | 25.12 | |  |
|  |  |  |  | **2-2** | 22.87 | | 24.02 | | 24.97 | | 24.08 | | 25.46 | 24.63 | 22.62 | 23.09 | | 25.55 | |  |
|  |  |  |  | **2-2** | 22.88 | | 24.12 | | 24.96 | | 24.18 | | 25.09 | 24.66 | 22.65 | 23.21 | | 25.32 | |  |
|  |  |  |  | **2-2** | 23.11 | | 24.12 | | 24.78 | | 24.24 | | 25.34 | 24.97 | 22.76 | 23.24 | | 25.72 | |  |
|  |  |  |  | **2-3** | 22.76 | | 24.04 | | 24.97 | | 24.77 | | 24.55 | 24.68 | 22.60 | 23.27 | | 25.32 | |  |
|  |  |  |  | **2-3** | 22.88 | | 24.28 | | 24.77 | | 24.57 | | 24.71 | 24.62 | 22.61 | 23.40 | | 25.21 | |  |
|  |  |  |  | **2-3** | 22.92 | | 24.47 | | 24.62 | | 24.38 | | 24.82 | 24.79 | 22.85 | 23.25 | | 25.36 | |  |
|  |  | **S3** | | **3-1** | 23.79 | | 24.73 | | 25.79 | | 24.44 | | 25.25 | 26.62 | 23.31 | 23.32 | | 26.07 | |  |
|  |  |  |  | **3-1** | 23.59 | | 24.80 | | 25.46 | | 24.46 | | 25.34 | 26.04 | 23.31 | 23.34 | | 26.00 | |  |
|  |  |  |  | **3-1** | 23.71 | | 24.80 | | 26.03 | | 24.81 | | 25.73 | 26.27 | 23.58 | 23.36 | | 26.28 | |  |
|  |  |  |  | **3-2** | 23.55 | | 24.57 | | 25.72 | | 24.57 | | 26.67 | 24.55 | 23.33 | 23.30 | | 28.97 | |  |
|  |  |  |  | **3-2** | 23.43 | | 25.04 | | 25.66 | | 24.73 | | 26.90 | 25.96 | 23.38 | 23.31 | | 29.06 | |  |
|  |  |  |  | **3-2** | 23.52 | | 24.74 | | 25.95 | | 24.70 | | 26.53 | 25.87 | 23.32 | 23.65 | | 29.11 | |  |
|  |  |  |  | **3-3** | 23.83 | | 25.00 | | 25.56 | | 24.70 | | 25.28 | 25.93 | 23.32 | 23.42 | | 26.31 | |  |
|  |  |  |  | **3-3** | 23.70 | | 24.79 | | 25.61 | | 24.83 | | 25.29 | 25.55 | 23.25 | 23.87 | | 26.39 | |  |
|  |  |  |  | **3-3** | 23.85 | | 25.14 | | 25.86 | | 25.27 | | 25.52 | 25.55 | 23.41 | 23.87 | | 26.43 | |  |
|  |  | **S4** | | **4-1** | 26.25 | | 26.11 | | 27.36 | | 26.26 | | 26.99 | 26.17 | 25.41 | 25.17 | | 27.91 | |  |
|  |  |  |  | **4-1** | 26.29 | | 26.24 | | 27.87 | | 26.15 | | 26.64 | 26.14 | 25.40 | 25.26 | | 28.02 | |  |
|  |  |  |  | **4-1** | 26.35 | | 26.02 | | 28.10 | | 26.36 | | 27.19 | 26.25 | 25.62 | 25.28 | | 27.96 | |  |
|  |  |  |  | **4-2** | 26.25 | | 26.09 | | 27.88 | | 26.28 | | 27.24 | 26.19 | 25.49 | 25.89 | | 27.10 | |  |
|  |  |  |  | **4-2** | 26.24 | | 26.10 | | 27.40 | | 26.33 | | 27.08 | 26.44 | 25.45 | 26.18 | | 27.03 | |  |
|  |  |  |  | **4-2** | 26.61 | | 26.17 | | 27.67 | | 26.47 | | 27.32 | 26.33 | 25.82 | 25.93 | | 27.18 | |  |
|  |  |  |  | **4-3** | 26.52 | | 26.37 | | 27.84 | | 26.64 | | 27.21 | 26.16 | 25.48 | 26.15 | | 28.10 | |  |
|  |  |  |  | **4-3** | 26.42 | | 26.24 | | 28.05 | | 26.54 | | 27.36 | 26.18 | 25.39 | 25.96 | | 28.12 | |  |
|  |  |  |  | **4-3** | 26.56 | | 26.71 | | 28.25 | | 26.88 | | 27.28 | 26.24 | 25.78 | 25.90 | | 28.51 | |  |
| **‘NJC108’** | | **S1** | | **1-1** | 22.49 | | 24.33 | | 27.00 | | 23.95 | | 24.62 | 25.34 | 22.70 | 23.12 | | 25.25 | |  |
|  |  |  |  | **1-1** | 22.41 | | 24.30 | | 26.85 | | 24.09 | | 24.41 | 25.14 | 22.67 | 23.07 | | 25.19 | |  |
|  |  |  |  | **1-1** | 22.64 | | 24.72 | | 26.77 | | 23.96 | | 24.36 | 25.27 | 22.58 | 23.56 | | 25.24 | |  |
|  |  |  |  | **1-2** | 22.66 | | 24.33 | | 26.83 | | 24.48 | | 24.52 | 24.31 | 22.34 | 23.12 | | 25.27 | |  |
|  |  |  |  | **1-2** | 22.58 | | 24.47 | | 26.81 | | 24.30 | | 24.65 | 24.12 | 22.49 | 23.04 | | 25.19 | |  |
|  |  |  |  | **1-2** | 22.81 | | 24.51 | | 26.90 | | 23.77 | | 25.16 | 24.14 | 22.68 | 22.94 | | 25.20 | |  |
|  |  |  |  | **1-3** | 22.82 | | 24.16 | | 26.72 | | 24.13 | | 24.15 | 24.26 | 23.07 | 23.56 | | 25.71 | |  |
|  |  |  |  | **1-3** | 22.95 | | 24.09 | | 26.80 | | 24.07 | | 24.22 | 24.04 | 22.90 | 23.36 | | 25.60 | |  |
|  |  |  |  | **1-3** | 22.81 | | 24.35 | | 26.86 | | 24.42 | | 24.11 | 24.23 | 22.86 | 23.46 | | 25.59 | |  |
|  |  | **S2** | | **2-1** | 32.72 | | 27.05 | | 24.65 | | 26.49 | | 27.76 | 27.61 | 26.26 | 26.18 | | 27.01 | |  |
|  |  |  |  | **2-1** | 33.10 | | 27.86 | | 25.10 | | 26.60 | | 27.58 | 27.67 | 26.53 | 26.14 | | 27.09 | |  |
|  |  |  |  | **2-1** | 32.76 | | 27.53 | | 25.82 | | 26.78 | | 27.20 | 27.28 | 26.34 | 26.88 | | 26.92 | |  |
|  |  |  |  | **2-2** | 33.68 | | 27.03 | | 26.18 | | 26.67 | | 27.00 | 26.54 | 26.13 | 26.56 | | 26.72 | |  |
|  |  |  |  | **2-2** | 33.57 | | 27.48 | | 27.13 | | 26.48 | | 27.53 | 26.09 | 26.16 | 26.64 | | 27.38 | |  |
|  |  |  |  | **2-2** | 33.86 | | 27.36 | | 27.14 | | 26.84 | | 28.29 | 26.24 | 26.16 | 26.25 | | 26.91 | |  |
|  |  |  |  | **2-3** | 33.62 | | 25.21 | | 27.20 | | 26.32 | | 26.65 | 26.61 | 26.48 | 26.74 | | 26.82 | |  |
|  |  |  |  | **2-3** | 33.11 | | 25.42 | | 27.32 | | 26.37 | | 26.60 | 26.58 | 26.67 | 26.77 | | 26.74 | |  |
|  |  |  |  | **2-3** | 33.93 | | 25.17 | | 27.14 | | 26.56 | | 26.83 | 26.67 | 26.71 | 26.99 | | 26.68 | |  |
|  |  | **S3** | | **3-1** | 23.05 | | 25.03 | | 26.23 | | 24.19 | | 24.53 | 25.71 | 24.43 | 24.17 | | 26.00 | |  |
|  |  |  |  | **3-1** | 23.39 | | 25.01 | | 26.30 | | 24.10 | | 24.36 | 25.55 | 24.36 | 24.10 | | 26.27 | |  |
|  |  |  |  | **3-1** | 23.21 | | 25.04 | | 26.27 | | 24.15 | | 24.49 | 25.75 | 24.49 | 24.20 | | 25.86 | |  |
|  |  |  |  | **3-2** | 23.27 | | 25.38 | | 26.01 | | 24.22 | | 24.02 | 25.69 | 24.27 | 24.08 | | 26.32 | |  |
|  |  |  |  | **3-2** | 23.40 | | 25.12 | | 25.91 | | 24.22 | | 23.88 | 25.73 | 24.24 | 23.95 | | 26.20 | |  |
|  |  |  |  | **3-2** | 23.30 | | 24.97 | | 26.09 | | 24.42 | | 24.03 | 25.97 | 24.25 | 24.05 | | 26.56 | |  |
|  |  |  |  | **3-3** | 22.55 | | 25.13 | | 26.46 | | 24.29 | | 24.05 | 25.43 | 24.83 | 24.01 | | 26.10 | |  |
|  |  |  |  | **3-3** | 22.70 | | 25.30 | | 26.37 | | 24.33 | | 24.05 | 25.41 | 24.70 | 23.86 | | 26.07 | |  |
|  |  |  |  | **3-3** | 22.50 | | 25.30 | | 26.13 | | 24.71 | | 23.93 | 25.58 | 24.78 | 24.00 | | 26.14 | |  |
|  |  | **S4** | | **4-1** | 23.61 | | 25.54 | | 25.64 | | 23.36 | | 23.58 | 27.13 | 23.65 | 23.66 | | 25.85 | |  |
|  |  |  |  | **4-1** | 23.44 | | 25.55 | | 25.59 | | 23.36 | | 23.54 | 26.95 | 23.45 | 23.57 | | 25.73 | |  |
|  |  |  |  | **4-1** | 23.43 | | 26.04 | | 25.71 | | 23.35 | | 23.33 | 27.53 | 24.17 | 23.51 | | 25.61 | |  |
|  |  |  |  | **4-2** | 22.60 | | 25.24 | | 25.41 | | 23.07 | | 23.23 | 26.27 | 24.11 | 23.39 | | 26.31 | |  |
|  |  |  |  | **4-2** | 22.49 | | 25.46 | | 25.47 | | 22.94 | | 23.28 | 26.18 | 24.10 | 23.37 | | 26.28 | |  |
|  |  |  |  | **4-2** | 22.86 | | 25.43 | | 25.56 | | 23.31 | | 23.47 | 26.43 | 24.88 | 23.45 | | 26.29 | |  |
|  |  |  |  | **4-3** | 22.82 | | 25.19 | | 25.37 | | 23.93 | | 23.36 | 26.65 | 23.73 | 23.54 | | 25.69 | |  |
|  |  |  |  | **4-3** | 23.21 | | 25.27 | | 25.35 | | 24.08 | | 23.27 | 26.59 | 23.71 | 23.39 | | 25.77 | |  |
|  |  |  |  | **4-3** | 22.99 | | 25.52 | | 25.51 | | 24.30 | | 23.54 | 26.82 | 23.98 | 23.64 | | 26.02 | |  |
